# *BrUFO* positively regulates the infection of Chinese cabbage by *Plasmodiophora brassicae*

**DOI:** 10.3389/fpls.2023.1128515

**Published:** 2023-03-09

**Authors:** Bo Zhang, Hui Feng, Wenjie Ge, Xinlei Wang, Jing Zhang, Ruiqin Ji

**Affiliations:** Liaoning Key Laboratory of Genetics and Breeding for Cruciferous Vegetable Crops, College of Horticulture, Shenyang Agricultural University, Shenyang, Liaoning, China

**Keywords:** Chinese cabbage, BrUFO, *Plasmodiophora brassicae*, VIGS, Yeast two-hybrid

## Abstract

**Introduction:**

Chinese cabbage is one of the most important vegetable crops in China. However, the clubroot disease caused by the infection of *Plasmodiophora brassicae (P. brassicae)* has seriously affected the yield and quality of Chinese cabbage. In our previous study, *BrUFO* gene was found to be significantly up-regulated in diseased roots of Chinese cabbage after inoculation with *P. brassicae. UFO (UNUSUAL FLORAL ORGANS)* have the properties of substrate recognition during ubiquitin-mediated proteolysis. A variety of plant can activate immunity response through the ubiquitination pathway. Therefore, it is very important to study the function of *UFO* in response to *P. brassicae*.

**Methods:**

In this study, The expression pattern of *BrUFO* Gene was measured by qRT-PCR and *In situ* Hybridization (ISH). The expression location of *BrUFO* in cells was determined by subcellular localization. The function of *BrUFO* was verified by Virus-induced Gene Silencing (VIGS). proteins interacting with BrUFO protein were screened by yeast two-hybrid.

**Results:**

Quantitative real-time polymerase chain reactions (qRT-PCR) and in situ hybridization analysis showed that expression of *BrUFO* gene in the resistant plants was lower than that in susceptible plants. Subcellular localization analysis showed that *BrUFO* gene was expressed in the nucleus. Virus-induced gene silencing (VIGS) analysis showed that silencing of *BrUFO* gene reduced the incidence of clubroot disease. Six proteins interacting with BrUFO protein were screened by Y_2_H assay. Two of them (Bra038955, a B-cell receptor-associated 31-like protein and Bra021273, a GDSL-motif esterase/acyltransferase/lipase Enzyme) were confirmed to strongly interact with BrUFO protein.

**Discussion:**

*BrUFO* gene should be a key gene of chinese cabbage against the infection of *P. brassicae. BrUFO* gene silencing improves the resistance of plants to clubroot disease. BrUFO protein may interact with CUS2 to induce ubiquitination in PRR-mediated PTI reaction through GDSL lipases, so as to achieve the effect of Chinese cabbage against the infection of *P. brassicae.*

## 1 Introduction

Clubroot disease is a worldwide soil-borne disease caused by *Plasmodiophora brassicae* Woron, which mainly infects Chinese cabbage, pakchoi, cabbage, radish, broccoli, mustard, rape and other cruciferous plants (Dixon, 2009; Wei et al., 2016; Yang et al., 2020). In recent years, clubroot disease has rapidly expanding around the world, leading to a significant decline in the yield and quality of Chinese cabbage, and has become one of a main diseases (Long et al., 2019; Zhang et al., 2020). It is safe, efficient and economical to cultivate resistant varieties by using resistance genes, and it is also one of the important ways to fundamentally solve the problems of clubroot disease (Sun et al., 2005).

In our previous study, a gene was found significantly up-regulated expressed on infected Chinese cabbage roots by *P. brassicae*, with a more than 5 times different expression between inoculated and uninoculated roots. Bioinformatics analysis showed that it has 91.26% sequence homology with *Arabidopsis UFO* (*Unusual Floral Organs*), so we named it *BrUFO*. UFO, as a part of the ubiquitin E3 complex, specifically degrades proteins negatively regulated by AP3 (APETALA 3) and those proteins that control normal growth in flower primordia (Lee et al., 1997). *UFO* genes are involved in regulating floral meristem and floral organ characteristics during flower development (Lee et al., 1997). In addition, *UFOs* may also play a role in early petal formation (Durfee et al., 2003). Therefore, UFO regulates multiple stages of flower development in the SCF complex.

UFO is an F-box protein containing F-box motifs and have the properties of substrate recognition during ubiquitin-mediated proteolysis. These proteins play an important role in many physiological processes such as cell time-phase conversion, signal transduction and development (Hershko, 1996). F-box proteins are SCF (SKP-Cullin-F-box) E3 ubiquitin ligase complexes that mediate ubiquitination and subsequent proteasome degradation of the target protein. Ubiquitination is one of the biochemical reactions that regulate the degradation of egg white matter in plants, and its functions are related to plant growth, development, biological stress and abiotic stress. Plants defend themselves against pathogen infections through a variety of mechanisms (Wielkopolan and Obrepalska-Steplowska, 2016). In recent years, scientists have found that several key ubiquitination enzymes (E1, E2, E3) are involved in plant disease resistance (Kravtsova-Ivantsiv and Ciechanover, 2011). A variety of plant can activate PTI (pamp to trigger immunity) response through ubiquitination pathway to inhibit pathogen infection (Sandra and Birgit, 2009). Many studies have shown that ubiquitination plays an important role in the PRR (People Recombinant Protein)-mediated PTI response. Ubiquitin-dependent protein degradation pathway is an effective regulatory mechanism for protein degradation in all organisms. It has been found that the infection of some twin viruses can interfere with the activity of E3 ubiquitin ligase through JA and GA pathways, thus providing favorable conditions for infection of the pathogen (Lozano-Durán et al., 2011; Ji et al., 2017; Jia et al., 2016; Kushwaha et al., 2017; Li et al., 2019). In addition, GDSL protein family plays an important role in jasmonic acid (JA) antagonistic SA signaling pathway. Many studies have proved that some GDSL proteins can enhance plant resistance to some pathogens through the SA resistance pathway (Hong et al., 2008; Kwon et al., 2009; Kim et al., 2014; Gao et al., 2017). But there are no reports about the direct relationship between *UFO* and disease resistance. Therefore, it is very important to study the function of *UFO* in response to *P. brassicae*.

In this study, the function and possible regulatory mechanism of Chinese cabbage *BrUFO* in response to the infection of *P. brassicae* was analyzed. These results provide a theoretical basis for using *BrUFO* gene to improve the resistance of Chinese cabbage to *P. brassicae.*

## 2 Materials and methods

### 2.1 Material

Plant materials used in this project were susceptible variety ‘SN742’ and resistant variety ‘SN205’of Chinese cabbage. The pathogen used in this study was physiological race NO.4 of *P. brassicae* isolated in previous work (Lv et al., 2021). The plants were cultured at 23°C with illumination for 12 h per day and 60% humidity. Roots samples were collected on the 14^th^ day after inoculation with *P. brassicae* (when zoospores of *P. brassicae* can be observe d in the root hairs under the ordinary light microscope), and on the 40^th^ day after inoculation with *P. brassicae* (when obvious club symptoms are appear on roots), and then stored at -80°C after flash-freezing in liquid nitrogen.

### 2.2 Method

#### 2.2.1 qRT-PCR analysis of *BrUFO*

In this study, the expression level of *BrUFO* in the roots of susceptible and resistant materials at different stages after inoculation with *P. brassicae* was quantitatively measured by qRT-PCR. In detail, total RNA was extracted using a Steady Pure Plant RNA Extraction Kit (Ecri Bio, Hunan, China), and RNA was reverse-transcribed into cDNA using HiScript 1st Strand cDNA Synthesis Kit (Vazyme, Nanjing, China). Specific primer P1 was designed according to the coding sequence (CDS) of Chinese cabbage *BrUFO*, and the *BrActin* gene was used as the internal control (the primers of P0 are shown in **Table 1**). qRT-PCR analysis was performed on the QuantStudio™ 6 Flex Real-Time PCR System (Life Technologies™, China) with ChamQ Universal SYBR qPCR Master Mix (Vazyme, Nanjing, China).

**Table 1:** Primer sequences related to *BrUFO*.

Primer names	Forward/Reverse primers	Primer sequences
**P0**	*BrActin*-F	ATCTACGAGGGTTATGCT
	*BrActin*-R	CCACTGAGGACGATGTTT
**P1**	*BrUFO*-F	TGAAATCAGATGGTATCGTC
	*BrUFO*-R	AGCCTTCCTCTGCTCTCTAA
**P2**	*BrUFO*-P-F	ACAAGCTTGCATGCCTGCAGGCTACCTCCTTCGCTTC
	*BrUFO*-K-R	CGGAATTCGAGCTCGGTACCGTTTGCTCCTCCTCCAC
**P3**	*BrUFO*-GFP-F	CGCCACTAGTGGATCCATGGAAACAAATATGTTCATCAATAAC
	*BrUFO*-GFP-R	GAGCGGTACCCTCGAGACAGGCTCCAGGAAATGGAAGTGCTAAC
**P4**	pTRV2-*BrUFO*-F	GGAGGCCAGTGAATTCATGGAAACAAATATGTTCATCAATA
	pTRV2-*BrUFO*-R	GAGCGGTACCCTCGAGACAGGCTCCAGGAAATGGAAGTGCTAAC
**P5**	pGBKT7-*BrUFO*-F	AGGAGGACCTGCATATGATGGAAACAAATATGTTCATCAATAAC
	pGBKT7-*BrUFO*-R	TCGACGGATCCCCGGGACAGGCTCCAGGAAATGGAAGTGCTAAC

The underlined part is the restriction site of restriction enzyme.

The study was arranged with three replicates for each different treatment. Duncan multi-range test (α = 0.05) was used to evaluate the expression levels of *BrUFO*. Data were analyzed by SPSS 11.5 (IBM, Armonk, NY, USA), and OriginPro 7.5 (OriginLab Corp., Northampton, MA, USA) was used to make graphs.

#### 2.2.2 *In situ* Hybridization analysis of *BrUFO*

*In situ* Hybridization (ISH) was performed to verify the results of qRT-PCR. For probes construction: specific primer P2 containing *Pst*I and *Kpn*I restriction sites were designed according to the CDS sequence of *BrUFO* (**Table 1**), and a 263 bp sequence was amplified and purified, then digested by *Pst*I and *Kpn*I. The digested product was ligated to the pSPT18 vector, and the resulting construct transformed, amplified, digested by *Pst*I or *Kpn*I. Probes were constructed using a DIG RNA Labeling Kit (SP6/T7; Roche, Basel, Switzerland) for *in vitro* transcription. For preparation of paraffin sections of root tissues: the roots of susceptible and resistant materials at different stages after inoculation with *P.brassicae* were fixed with 4% Paraformaldehyde solution (PFA) (Rnase-free) solution. Uninoculated roots were used as control. Evacuate the samples with a vacuum pump until sanking into the bottom of the fixing solution, and then submerged in paraffin overnight at 4°C (Ji et al., 2017). Fixed the embedded samples on the slicer for slicing (cut at least three slices each treatment), and paraffin sections containing samples were placed in DEPC-H_2_O at 42°C to attach to slides, and dried overnight. Paraffin sections were dewaxed in a series of ethanol:xylene mixtures (100% xylene, 1:3 ethanol:xylene, 1:1 ethanol:xylene, 3:1 ethanol:xylene, and 100% ethanol) for 5 minutes in each mixture.

The dewaxed plant sections hybridized with *BrUFO* probe, and then were stained and observed under optical microscope (ECLIPSE Ci-L, Nikon) following Ji et al. (2017).

#### 2.2.3 Subcellular localization analysis of *BrUFO*

The expression location of *BrUFO* in cells was determined by subcellular localization. The full-length cDNA of *BrUFO* was amplified using primer P3 with *BamH*I and *Xho*I restriction sites (**Table 1**) from the roots of susceptible materials, and then ligated to pBWA(V)BS-GFP to obtained pBWA(V)BS-*BrUFO*-GFP. pBWA(V)BS-*BrUFO*-GFP was transformed into agrobacterium GV3101, and then cultured overnight, centrifuged. The cell pellet was resuspended in infection solution (containing 10 mM MgCl_2_, 10 mM MES, 15 μL AS, pH = 5.6). The obtained suspension (OD600 = 1.0) was injected into 4-week-old tobacco leaves. After 2 days of dark culture, the leaves were observed under a laser confocal microscope (TCS SP8, Leica, Germany).

#### 2.2.4 Function verification of *BrUFO* in Chinese cabbage by virus-induced gene silencing

The function of BrUFO was verified by Virus-induced Gene Silencing (VIGS). In detail, a 300bp cDNA fragment of *BrUFO* was connected to pTRV2 to constructed pTRV2-*BrUFO* silenced vector. pTRV2-*00* (empty vector) and pTRV2- *BrUFO* were transferred into *A. tumefaciens* strain GV3101 separately using the freeze-thaw method (Zhou et al., 2021). The infection solution containing vectors pTRV2-*00*, pTRV2- *BrUFO* were mixed respectively with the infection solution containing pTRV1 vector (1:1, vol/vol) to constructed pTRV1+pTRV2-*00* or pTRV1+pTRV2- *BrUFO* mixed infection solution. Germinated seeds were treated with above two kinds of mixed infection solution respectively by the agrobacterium vacuum infiltration method (Yue et al., 2021), wild-type (untreated) plants served as controls. 60 seeds were infected in each group (the experiment was repeated three times). The seeds treated with the mix infection solution were cultured in a 25 °C incubator with a 16 h light/8 h dark cycle. When the gene silencing was confirmed, the roots were inoculated with *P. brassicae* suspension. When the typical swelling symptom of clubroot disease was obvious, the expression level of *BrUFO* was measured by qRT-PCR with a specific primer pair P4 (**Table 1**). All treatments were performed in triplicate with five randomly selected plants per treatment for each replicate. Duncan’s multiple range tests (α=0.05) were conducted to evaluate significant differences. Data were analyzed by SPSS 11.5 (IBM, Armonk, NY, USA), and OriginPro 7.5 (OriginLab Corp., Northampton, MA, USA) was used to produce graphs. At the same time, when clubroot symptom was showed, the incidence and level of clubroot disease were analyzed using a completely random design with three replicates and 30 seedlings per experimental unit. The infection progress of inoculated seedlings was analyzed by Fisher’s least significant difference test at a significance threshold of p <0.05.

#### 2.2.5 Yeast two-hybrid (Y_2_H) analysis

In order to screen the interaction proteins of BrUFO, the full-length sequence of *BrUFO* with *Nde*I and *Xma*I restriction sites were amplificated with primer P5 (**Table 1**) and connected to pGBKT7 to obtained recombinant vector pGBKT7-*BrUFO*. Self-activation and toxicity tests of the PGBKT7-*BrUFO* were performed to exclude the self-expression influence.

Y_2_H screening was conducted using yeast mating according to the Matchmaker Gold Y_2_H System user manual (Clontech, USA). Strains carrying PGBKT7-*BrUFO* were mixed with the library strain and screened on SD/-LEU/- TRP/X-α-Gal/AbA (DDO/X/A) medium. All single blue colonies were selected and streaked on SD/-ADE/-HIS/- LEU/-TRP/X-α-Gal/AbA (QDO/X/A) medium. Positive blue colonies were assessed by PCR, and sequenced. The sequencing results were compared with the BLAST database using BRAD (http://brassicadb.cn/#/) to obtain potential interaction proteins of BrUFO.

#### 2.2.6 Point-to-point interaction verification between candidate proteins and BrUFO

The full-length cDNA sequences of candidate proteins were cloned and connected to PGADT7 to construct PGADT7 recombinant vectors respectively. Each PGADT7 recombinant vector and PGBKT7-*BrUFO* recombinant vector were co-transfected into Y_2_H Gold competent yeast cells respectively, and then cultivated in DDO medium at 30°C for 72-96 h. Selected the well-grown monocolones, and resuspensed them with 30 μL of sterile ultra-pure water. Incubated them on the QDO/A/X medium upside down at 30°C for 72-96 h, and observed the growth of colones.

## 3 Results

### 3.1 The expression pattern analysis of *BrUFO* gene

**Figure 1:**
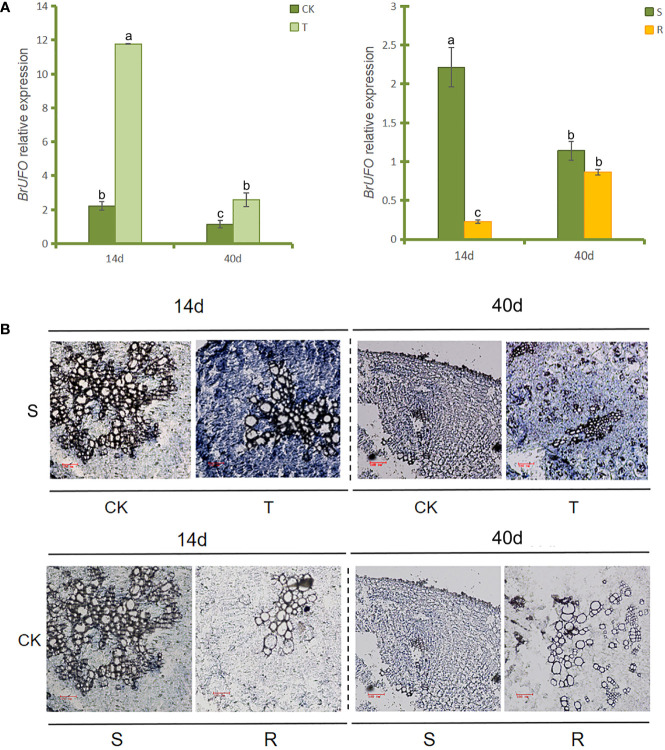
*BrUFO* expression patterns. **(A)** qRT-PCR analysis of *BrUFO*. Results are mean ± standard deviation (n = 3). Each set of data represents the mean of its three replicates, and the error line represents the standard deviation of its three replicates. Letters indicate significant differences at *p ≤*0.05 according to Duncan’s multiple range test. **(B)** *In situ* hybridization analysis of *BrUFO*. Roots samples were collected on the 14^th^ day after inoculation with *P. brassicae* (when zoospores of *P. brassicae* can be observe d in the root hairs under the ordinary light microscope), and on the 40^th^ day after inoculation with *P. brassicae* (when obvious club symptoms are appear on roots); S, susceptible material of Chinese cabbage susceptible material’SN742’; R, Chinese cabbage resistant material ‘SN205’; CK, control roots not inoculated with *Plasmodiophora brassicae*; T, treated roots inoculated with *Plasmodiophora brassicae*.

qRT-PCR results of *BrUFO* in the roots of Chinese cabbage showed that the expression level of *BrUFO* in the inoculation-treated group (T) was higher than that in the uninoculated control group (CK) (**Figure 1A**). In addition, the expression level of *BrUFO* was higher in susceptible materials than that in resistant materials. Thus, it was concluded that low expression of *BrUFO* likely enhances the resistance of plants to *P. brassicae*.

*In situ* hybridization was used to verify the expression patterns of *BrUFO* in the roots of Chinese cabbage. The results revealed blue hybridization signals were showed in inoculated roots in both sampling periods (**Figure 1B**). Furthermore, the signal of *BrUFO* was stronger in susceptible materials than that in resistant materials when the plants was not been infected by *P. brassicae*, but low expression of *BrUFO* makes the difference indistinguishable by naked eye. Thus, the results of *in situ* hybridization were consistent with that of qRT-PCR.

**Figure 2:**
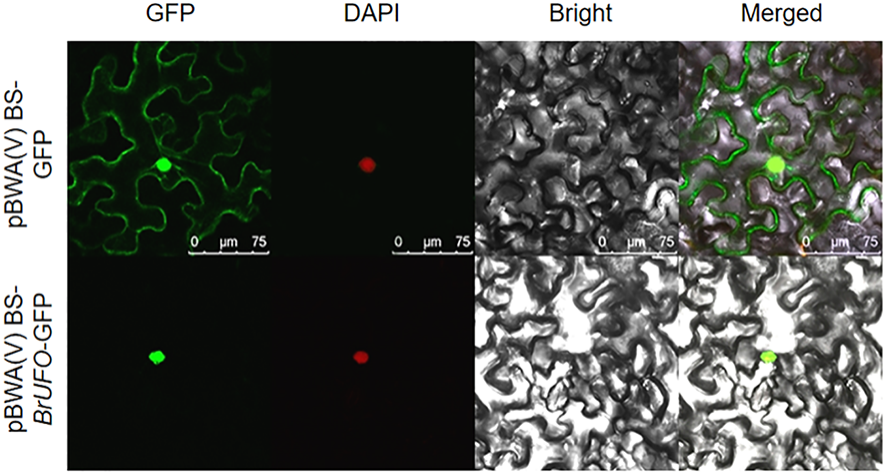
Subcellular localization of *BrUFO.* The green fluorescence signal is from GFP; the red fluorescence signal is from DAPI; the yellow fluorescence signal is the result of superposition of green and red fluorescence signals.

UFO is an F-box protein (Samach et al., 1999; Wang et al., 2003), and some F-box proteins are located in the nucleus, such as VVF-Box5 of the grape F-Box family is mainly located in the nucleus of onion epidermal cells (Yu et al., 2018). The F-box protein of *P. euphratica* (Ren et al., 2017) and F-box family protein SIF-Box18 in millet (Huo et al., 2014) are also located in the nucleus. In order to clarify the action site of BrUFO in Chinese cabbage, subcellular localization was performed. The results showed that the leaves containing pBWA(V)BS-GFP (empty vector) showed fluorescence signals in the nucleus, cell membrane and cytoplasm. However, the leaves containing pBWA(V)BS-*BrUFO*-GFP recombinant vector only showed fluorescence signals in the nucleus. After staining with DAPI, the leaves containing pBWA(V)BS-GFP or pBWA(V)BS-*BrUFO*-GFP emitted a red signal on nucleus (**Figure 2**), which was the location of the GFP channel. Therefore, it was inferred that BrUFO was located in the nucleus. This result provided a basis for screening proteins potentially interacting with BrUFO from the Y_2_H nuclear library.

### 3.2 The functional validation of *BrUFO*

**Figure 3:**
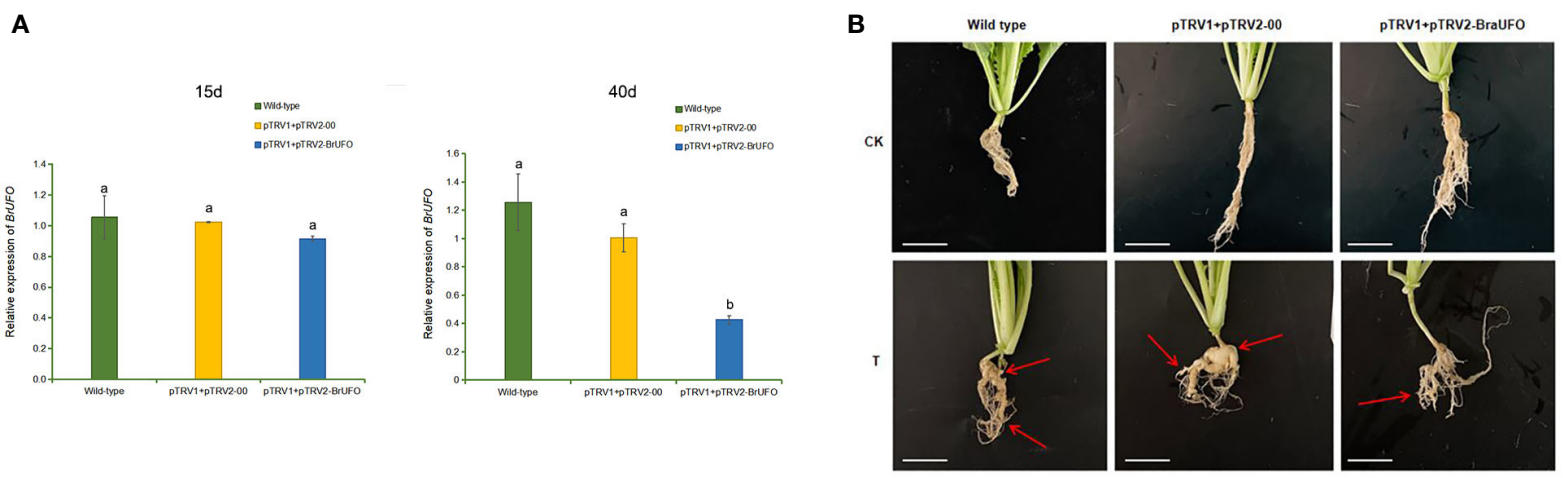
Functional validation of *BrUFO* in Chinese cabbage responses to infection by *Plasmodiophora brassicae.* **(A)** Expression of *BrUFO* in the roots of Chinese cabbage after VIGS treatment. Results are mean ± standard deviation (n = 3). Each set of data represents the mean of its three replicates, and the error line represents the standard deviation of its three replicates. Letters indicate significant differences at *p ≤*0.05 according to Duncan’s multiple range test. **(B)** Symptoms of clubroot disease in VIGS-treated roots on day 40 after VIGS treatment. CK, control plants not inoculated with *P. brassicae*; T, plants treated with *P. brassicae.* On the 40^th^ day after VIGS treatment (i.e. the 25^th^ day after inoculation with *P. brassicae*), obvious clubroot symptoms were showed on the taproots and fibrous roots of both control plants. To pTRV1+pTRV2-*BrUFO* plants, only mild swelling symptoms were evident on the fibrous roots, but not on taproots. Scale bar=2 cm.

The VIGS technique was used to verify the function of *BrUFO* in Chinese cabbage. The results showed that *BrUFO* began to be silenced on the 15^th^ day after pTRV1+pTRV2-*BrUFO* VIGS treatment, which showed as the expression levels of *BrUFO* in plants harbouring pTRV1+pTRV2-*BrUFO* were lower than in pTRV1+pTRV2-*00* (empty vector) and untreated wild-type plants. On the 25^th^ day after VIGS treatment, the expression of *BrUFO* was significantly down-regulated in plants harbouring pTRV1+pTRV2-*BrUFO*. When the expression level of *BrUFO* in pTRV1+pTRV2-*00* plants was set as 1, the expression levels in pTRV1+pTRV2-*BrUFO* plants was only 0.4, and the silencing efficiency was 60% (**Figure 3A**). On the 40^th^ day after VIGS treatment (i.e. the 25^th^ day after inoculation with *P. brassicae*), obvious swelling symptoms were showed on the taproots and fibrous roots of two control plants. To pTRV1+pTRV2-*BrUFO* plants, only mild swelling symptoms were evident on the fibrous roots, but not on taproots (**Figure 3B**). The average clubroot disease incidence (20 plants per treatment) of untreated, pTRV1+pTRV2-*00* and pTRV1+pTRV2-*BrUFO* treated plants were 11, 42.25 and 7.25, respectively. And their average incidence rates were 35%, 85% and 15%, respectively (**Table 2**). pTRV1+pTRV2-*00* (empty vector) treatment significantly influenced the resistance of plants, while the silence of *BrUFO* restored the resistance of plants. These results suggest that the decrease *of BrUFO* expression can enhance the resistance of Chinese cabbage to *P. brassicae*.

**Table 2:** Disease indices and incidence rates of *BrUFO* Silenced Chinese cabbage infected with *P. brassicae*.

Group	Incidence rates(%)	Disease indices
Wild-type	35 ± 4.08^b^	10 ± 0.88^a^
pTRV1+pTRV2-*00*	85 ± 7.07^a^	41.25 ± 2.83^b^
pTRV1+pTRV2-*BrUFO*	15 ± 4.08^b^	6.25 ± 0.88^b^

Wild-type: untreated, naturally grown Chinese cabbage controls; pTRV1+pTRV2-00: empty vector treatment group; pTRV1+pTRV2-BrUFO: pTRV1+pTRV2-BrUFO treatment group. Results are mean ± standard deviation (n = 3) and ^a^ or ^b^ indicates significant differences at p ≤0.05 according to Duncan’s multiple range test.

### 3.3 Y_2_H screening for interaction proteins of BrUFO

**Figure 4:**
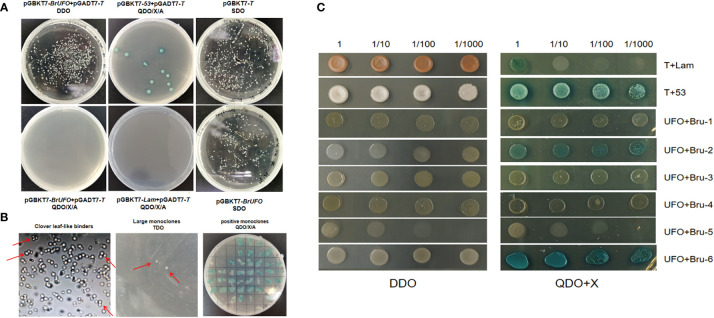
Yeast two hybrid test for BrUFO. **(A)** Self-activation analysis and toxicity testing of pGBKT7-*BrUFO.* **(B)** Screening of potentially interacting proteins with BrUFO from the yeast library. Left, Cloverleaf-like binders on TDO medium. Middle, Large monoclonal colonies in TDO transferred to QDO medium. Right, Positive interaction of proteins with BrUFO on DDO/X/A medium. **(C)** Validation of proteins interacting with BrUFO. Co-transformation results for proteins potentially interacting with BrUFO on DDO medium and QDO+X-α-Gal medium.

To assess the self-activation ability of bait vector PGBKT7-*BrUFO*, PGBKT7-*BrUFO* and pGADT7-*T* were co-transfected into Y_2_H competent cells. The results showed that white colonies could grow on DDO solid plates, but no colonies grew after single colonies were diluted and plated on QDO/A/X solid plates as well as negative controls (pGBKT7-*Lam* + pGADT7-*T*). However, positive controls (pGBKT7-*53* + pGADT7-*T*) yielded blue colonies on QDO/A/X solid plates (**Figure 4A**). These results indicate that the expressed protein of PGBKT7-*BrUFO* did not possess self-activation ability. The toxicity test results for bait vector PGBKT7-*BrUFO* showed that the growth of Y187 colonies containing PGBKT7-*BrUFO* were similar to those of colones containing PGBKT7-*T* on SDO solid plates (**Figure 4A**). These results proved that the pGBKT7-*BrUFO* protein was not toxic, and it could therefore be used for subsequent library screening.

When the Y_2_H yeast monoclone containing PGBKT7-*BrUFO* was hybridized with the Chinese cabbage yeast library induced by clubroot, typical cloverleaf-like binders were observed under the light microscope, and 366 large monoclonal colonies on TDO medium were picked and cultivated on QDO/X/A medium (**Figure 4B**). Positive monoclones on QDO medium were sequenced and subjected to BLAST to analyze the function using the NCBI database, and six proteins were eventually identified (**Table 3**); These proteins include: Bru-1, Haloacid dehalogenase-like hydrolase (HAD) superfamily protein (detected twice); Bru-2 (CUS2), GDSL-motif esterase/acyltransferase/lipase (detected once); Bru-3, UDP-N-acetylglucosamine transferase subunit ALG14-like protein (detected once); Bru-4, Component of the SPT module of the SAGA complex (detected once); Bru-5, Li-tolerant lipase 1 protein (detected once) and Bru-6, B-cell receptor-associated 31-like protein (detected once).

**Table 3:** Information for candidate proteins that interacted with BrUFO.

Gene number	Gene name	Homologous *Arabidopsis* thaliana	Description of interacting protein	Screening times	Identity
*Bru-1*	*Bra016945*	*AT2G41250*	Haloacid dehalogenase-like hydrolase (HAD) superfamily protein	2	99%
*Bru-2*	*Bra038955*	*AT5G33370 (CUS2)*	GDSL-motif esterase/acyltransferase/lipase. Enzyme group with broad substrate specificity that may catalyse *acyltransfer* or hydrolase reactions with lipid and non-lipid substrates. Mutants are defective in cuticle formation with reduced sepal cuticle ridge formation	1	99%
*Bru-3*	*Bra013293*	*AT4G18230*	UDP-N-acetylglucosamine transferase subunit ALG14-like protein	1	98%
*Bru-4*	*Bra011659*	*AT4G36080 (TRA1B)*	Component of the SPT module of the SAGA complex.	1	99%
*Bru-5*	*Bra040203*	*AT3G04290* *(ATLTL1)*	Li-tolerant lipase 1	1	99%
*Bru-6*	*Bra021273*	*AT1G48440*	B-cell receptor-associated 31-like protein	1	97%

### 3.4 The validation result of proteins interacting with *BrUFO*

To validate the interactions between candidate proteins and BrUFO, co-transfection results showed these colonies could grow on DDO medium (**Figure 4C**). However, on QDO/X medium, only PGADT7-*Bru-6*+PGBKT7-*BrUFO*, and PGADT7-*Bru-2*+PGBKT7-*BrUFO* yielded blue clones as well as pGADT7-*T* + PGBKT7-*53* (positive controls). Contrast with positive controls, stains harbouring other candidate proteins as well as pGAD7-*T* + PGBKT7-*T* (negative controls) did not grow colonies (**Figure 4C**). So it is determined that Bru-2 and Bru-6 interact with BrUFO.

## 4 Discussion

### 4.1 *BrUFO* should be a key gene of Chinese cabbage against the infection of *P. brassicae*

A series of changes will occur when plants are infected by pathogens, such as the changes of gene expression (Coll et al., 2011). For example, the infection of *magnaporthe oryzae* resulted in the increase of expression levels of *Bd4g02060*, *Bd1g21177*, *Bd5g06390*, *Bd3g41480*, and *Bd3g28280* in rice within 48 hours (Peng et al., 2021). Both *CsRSF1* and *CsRSF1* could respond to the *S. fuliginea* infection. In the early stage of *S. fuliginea* infection, the expression levels of *CsRSF1* and *CsRSF1* were higher in the resistant varieties, which had a positive regulatory effect on the *S. fuliginea* infection (Wang X et al., 2021). In this study, qRT-PCR and *in situ* hybridization results showed that *BrUFO* was significantly up-regulated expressed on the roots of Chinese cabbage infected by *P. brassicae*, and *BrUFO* expression levels were higher in susceptible materials than that in resistant materials (**Figure 1**).

Plant innate immune response system is mainly composed of PTI and ETI. Many studies have shown that ubiquitination plays an important role in the PRR-mediated PTI response (Sandra and Birgit, 2009). Some F-Box proteins function in plant defence responses dependent on ubiquitin. UFO is an F-box protein (Samach et al., 1999; Wang et al., 2003), and some F-Box proteins, such as Coronatine insensitive 1 (COI1) and Suppressor of nim1-1 (SON1) have the function in plant defence responses such as disease resistance and insect resistance (Xie et al., 1998; Kim and Delaney, 2002). *Arabidopsis* SON1 is an F-box protein that negatively regulates the resistance of pathogenic downy fungi and leaf spot bacteria through a selective protein degradation pathway dependent on ubiquitin (Kim and Delaney, 2002). These results suggest that BrUFO as an F-box protein played role in different types of disease resistance in plants.

### 4.2 *BrUFO* silencing improves the resistance of plants to clubroot disease

VIGS is a rapid and efficient method for studying gene function. It has the advantages of a short cycle, simple operation, and the capacity for high-throughput, and it is independent of a mature transgenic system (Burch-Smith et al., 2004). It is widely used in plant growth and development, such as pistil development, flower development, fruit cracking, and ripening (Zhao et al., 2015; Qi et al., 2018), as well as gene function identification related to flower colour, organ formation (Wang P. et al., 2021), plant disease resistance and abiotic stress (Zhao et al., 2015; Liu et al., 2019), signal transduction, and metabolite synthesis and regulation. Currently, more than 30 vectors have been developed using VIGS technology. Among them, tobacco rattle virus (TRV) is the most widely used VIGS vector in many herbs and some woody plants (Ye et al., 2009; Annette and Matthias, 2010; Huang et al., 2012; Sahu et al., 2012; Jiang et al., 2014). In this study, pTRV1+pTRV2-*BrUFO* was firstly used to silence *BrUFO*. The results showed that *BrUFO* was effectively silenced, and the incidence of clubroot disease was reduced in silenced plants, suggesting that *BrUFO* silencing could improve the resistance of plants to *P. brassicae*.

### 4.3 Regulation mechanism of BrUFO in Chinese cabbage against *Plasmodiophora brassicae*

A useful way to explore intracellular signal transduction is to study interactions between proteins (Waadt et al., 2010). The Y_2_H system is widely used to identify and verify interactions between proteins (Zhang et al., 2021). In this study, Bru-6 (Bra021273) and Bru-2 (Bra038955) were screened and confirmed to interact with BrUFO. Bru-6 is a receptor-associated 31-like protein but little is known about it. Bru-2 is CUS2, a GDSL-like esterase/acyltransferase/lipase protein.

**Figure 5:**
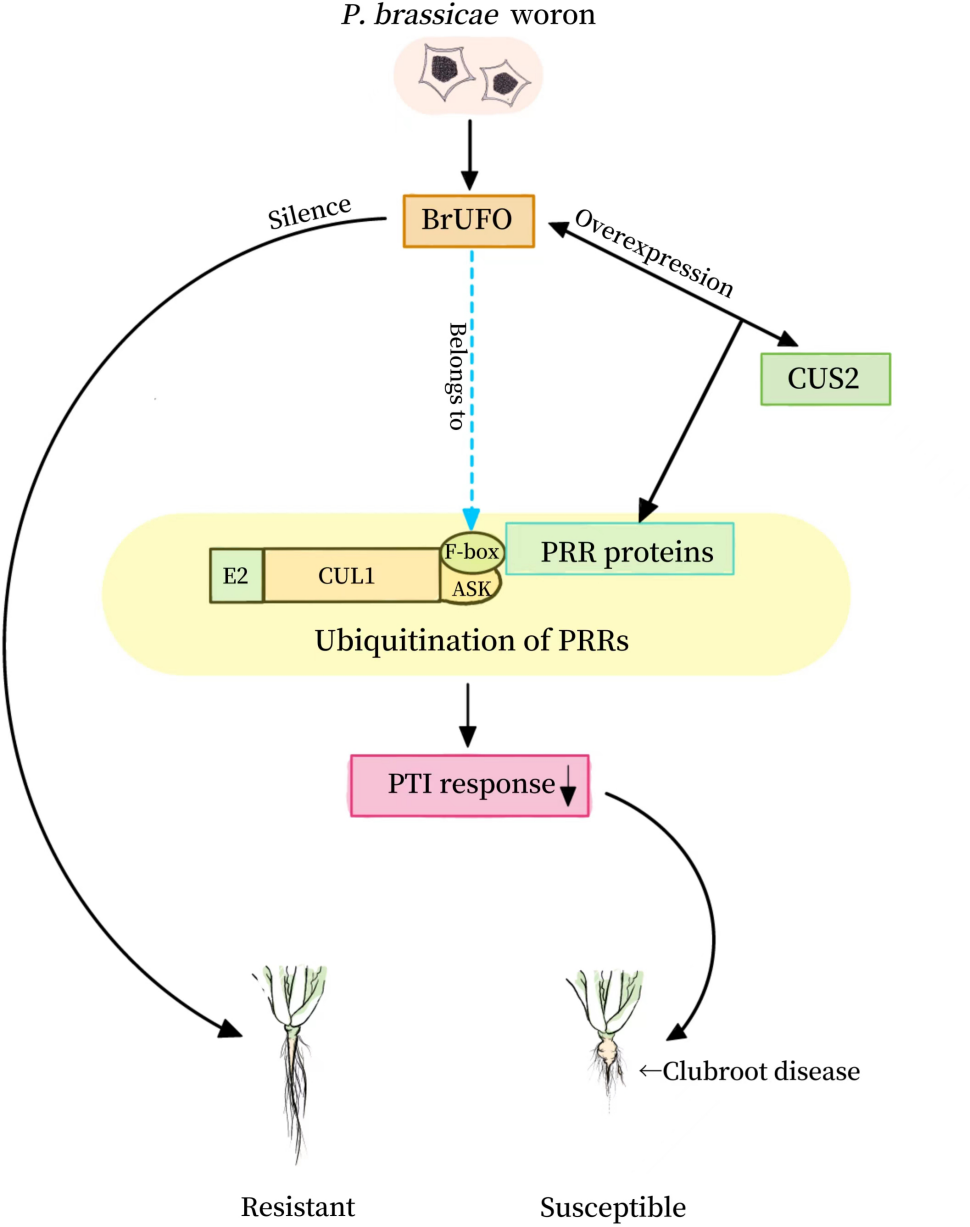
Role pattern of BrUFO in Chinese cabbage response to *Plasmodiophora brassicae*.

GDSL lipases play important roles in many physiological and biochemical processes such as plant growth and development (Takahashi et al., 2009; Dong et al., 2016; Ji et al., 2017), organ morphogenesis, lipid metabolism (Clauß et al., 2011; Ding et al., 2019a; Ding et al., 2019b) and stress reaction (Hong et al., 2008; Kwon et al., 2009; Lee et al., 2009; Kim et al., 2014). In addition, expression of GDSL lipase genes in some plants can be induced by pathogens, hormones such as salicylic acid (SA), ethylene and jasmonic acid (JA), and abiotic stress factors, suggesting that they may be involved in plant disease resistance and stress responses (Jakab et al., 2003; Lee and Cho, 2003; Hong et al., 2008; Kwon et al., 2009; Kim et al., 2014; Gao et al., 2017). GDSL protein family plays an important role in jasmonic acid (JA) antagonistic SA signaling pathway. In addition, many studies have proved that some GDSL proteins can enhance plant resistance to some pathogens through the SA resistance pathway (Hong et al., 2008; Kwon et al., 2009; Kim et al., 2014; Gao et al., 2017). The overexpression of *AtGDSL1* decreased the content of reactive oxygen species (ROS) and salicylic acid (SA), which enhanced the resistance of *Brassica napus* to *S. sclerotiorum* (Ding et al., 2020). The expression of two GDSL lipases, OsGLIP1 and OsGLIP2, in rice immune response were inhibited by pathogen infection and salicylic acid (SA) treatment. The simultaneous downregulation of *OsGLIP1* and *OsGLIP2* increased the resistance of rice to *Xoo* and *M. oryzae* (Gao et al., 2017). Therefore, it is speculated that BrUFO may interact with CUS2 to induce ubiquitination in PRR-mediated PTI reaction through GDSL lipases, so as to achieve the effect of Chinese cabbage against the infection of *P. brassicae* (**Figure 5**). However, this should be verified in follow-up studies.

## Data availability statement

The original contributions presented in the study are included in the article/supplementary material. Further inquiries can be directed to the corresponding author.

## Author contributions

BZ: Conceptualization, Methodology, Data curation, Writing-original draft, Formal analysis, Investigation, Writing-review & editing. HF: Formal analysis, Methodology, Data curation, Investigation. WG and XW: Formal analysis, Data curation. JZ: Formal analysis. RJ: Funding acquisition, Conceptualization, Formal analysis, Supervision, Writing-review & editing. All authors contributed to the article and approved the submitted version.
